# Intramolecular isopeptide but not internal thioester bonds confer proteolytic and significant thermal stability to the *S. pyogenes* pilus adhesin Spy0125

**DOI:** 10.1002/prot.24420

**Published:** 2013-10-17

**Authors:** Miriam Walden, Allister Crow, Miles D Nelson, Mark J Banfield

**Affiliations:** Department of Pathology, University of Cambridge, Tennis Court RoadCambridge CB2 1QP, United Kingdom

**Keywords:** *S. pyogenes* pili, intramolecular isopeptide bonds, internal thioester bonds, protein stability, circular dichroism, X-ray crystallography

## Abstract

*Streptococcus pyogenes* and other Gram-positive bacterial pathogens present long macromolecular filaments known as pili on their surface that mediate adhesion and colonization. These pili are covalent polymers, assembled by sortases. Typically, they comprise a putative adhesin at their tip, a backbone subunit present in multiple copies and a basal subunit that is covalently anchored to the peptidoglycan layer of the cell surface. The crystal structures of pilin subunits revealed the presence of unusual covalent linkages in these proteins, including intramolecular isopeptide and internal thioester bonds. The intramolecular isopeptide bonds in backbone pilins are important for protein stability. Here, using both the wild-type protein and a set of mutants, we assessed the proteolytic and thermal stability of the *S. pyogenes* pilus tip adhesin Spy0125, in the presence and absence of its intramolecular isopeptide and internal thioester bonds. We also determined a crystal structure of the internal thioester bond variant Spy0125^Cys426Ala^. We find that mutations in the intramolecular isopeptide bonds compromise the stability of Spy0125. Using limited proteolysis and thermal denaturation assays, we could separate the contribution of each intramolecular isopeptide bond to Spy0125 stability. In contrast, mutation in the internal thioester bond had a lesser effect on protein stability and the crystal structure is essentially identical to wild type. This work suggests that the internal thioester in Spy0125, although having a minor contributory role, is not required for protein stability and must have a different primary function, most likely mediating a covalent interaction with host cell ligands. Proteins 2014; 82:517–527. © 2013 The Authors Proteins: Structure, Function, and Bioinformatics Published by Wiley Periodicals, Inc.

## INTRODUCTION

The composition and assembly of long, flexible pili presented on the surface of Gram-positive bacterial pathogens have been extensively studied over the last ∼10 years. These pili are found on the surface of clinically relevant pathogens including *Bacillus cereus, Bacillus anthracis, Corynebacterium diphtheriae,*^1^ *Streptococcus agalactiae, Streptococcus pneumoniae*, *Streptococcus pyogenes*, and others (reviewed in ref. 2–5). They are immunogenic and knowledge of their structure and function offers the potential for novel approaches to the prevention of disease.

Gram-positive bacterial pili typically comprise repeats of a major pilin subunit, in a beads-on-a-string-type arrangement. They are assembled by the action of sortases, enzymes that catalyze the formation of intermolecular isopeptide bonds between the C-terminal carboxyl group of one subunit and a specific lysine side chain of the next subunit in the chain.^6^ In addition to the major subunit, pili also contain a distinct minor pilin at the tip of the structure and most, although not all, contain a second minor pilin at the base.^7^ Typically, the genes encoding pilus subunits and pilus-specific sortases are clustered in bacterial genomes.^8^ In three-component pili, the basal pilin is used to anchor the structure to the peptidoglycan layer (catalyzed by a generic “house-keeping” sortase), while in both three- and two-component pili the tip pilin most likely serves as a specific adhesin, presented away from the bacterial surface, that targets a host cell molecule of relevance to infection.^2^

In addition to intermolecular isopeptide bonds, domains of both major and minor pilins can contain intramolecular isopeptide bonds.^9^–^15^ These unusual bonds have been shown to confer increased stability to these domains, and likely act to support pilus function during harsh chemical and mechanical stresses that are encountered during infection.^13^,^16^,^17^

*Streptococcus pyogenes* (Group A *Streptococcus*) is a major human pathogen that can cause a variety of diseases, depending on the localization of infection.^18^ In M1 Group A *Streptococcus* (GAS) strain SF370, pili are comprised of three subunits, Spy0125 (minor tip pilin, also known as “Cpa”), Spy0128 (major pilin), and Spy0130 (minor basal pilin). These pili are essential for efficient adhesion of the bacteria to model host cells and to clinically relevant tissues.^19^,^20^

**Figure 1:**
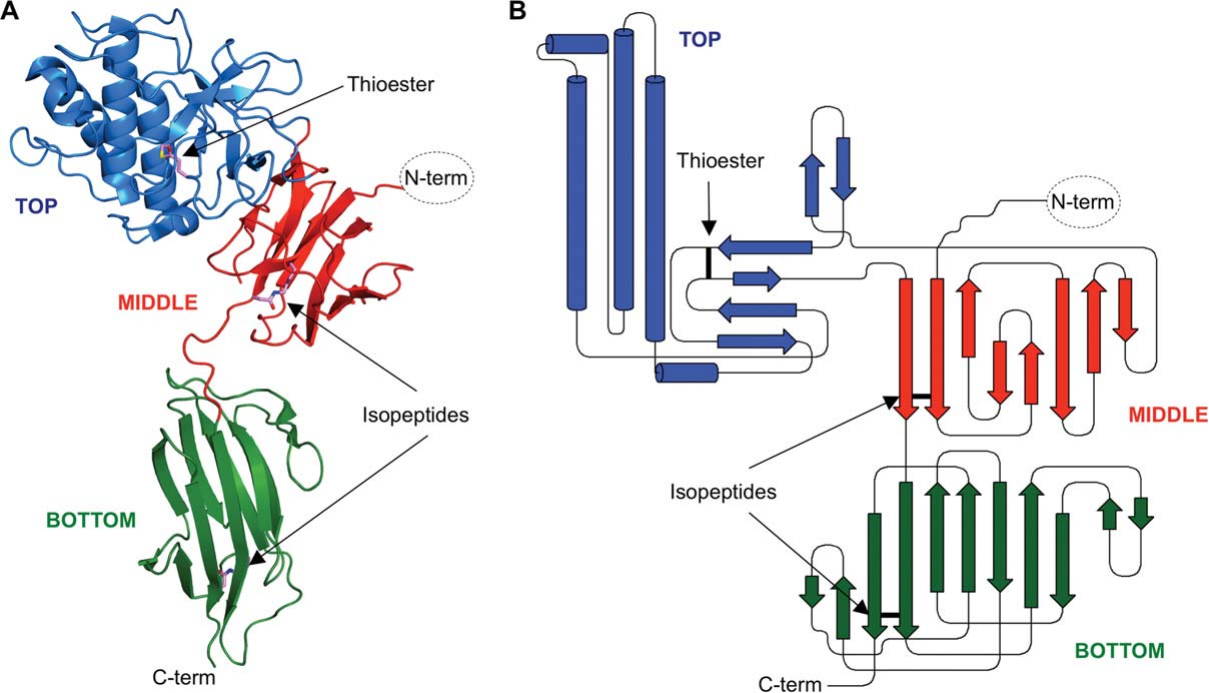
Overall structure and topology of Spy0125-CTR showing the positions of the intramolecular isopeptide and internal thioester bonds. (**A**) The structure of Spy0125-CTR is shown in ribbon form in blue (top domain), red (middle domain), and green (bottom domain). The positions of the internal thioester and the intramolecular isopeptides are indicated. (**B**) The topology diagram of Spy0125-CTR is colored as in (A). The positions of the thioester and isopeptides are shown as black bars.

To date, the crystal structures of two pilus tip adhesins have been determined, *S. pneumoniae* RrgA^13^ and the C-terminal region of *S. pyogenes* Spy0125 (Spy0125-CTR).^10^ Each of these proteins contains domains with structural homology to those found in major pilins, with three of these in RrgA and two in Spy0125-CTR. Most of these pilin domains contain stabilizing intramolecular isopeptide bonds (in Spy0125 these are between Asp595 and Lys297 and between Asn715 and Lys610 of the middle and bottom domains, respectively, Fig.1). However, RrgA contains a fourth domain that adopts a completely different fold, with homology to the human A3 domain of von Willebrand factor, a molecule shown to interact with collagens, and contains a MIDAS (metal ion-dependent adhesion) site.^13^ It is presumed that this region is responsible for the adhesive properties of the *S. pneumoniae* *rlrA* pilus to components of the host extracellular matrix.^13^ Also, the third domain defined in the crystal structure of Spy0125-CTR is not related to the fold found in major pilins. Intriguingly, the Spy0125-CTR structure revealed that this top domain contains an internal thioester bond between the side chains of Cys426 and Gln575^10^ (Fig.1). Such reactive thioester bonds have been implicated in covalent attachment of complement and complement-like proteins to target molecules.^21^–^23^ Bacteria engineered with a Spy0125^Cys426Ala^ mutation, designed to prevent the formation of the thioester, showed a 75% reduction in adhesion to model host cells.^10^ Therefore, the internal thioester in Spy0125 may mediate direct attachment to a host cell factor to promote bacterial adhesion during infection. However, alternative models where the thioester is involved in either protein folding or stability have yet to be discounted.

In this study, we set out to determine the roles of the intramolecular isopeptide bonds and internal thioester bond in the stability of the Spy0125. We have prepared variant Spy0125-CTR proteins (based on the stable, functional construct used for protein structure determination) that lack either the internal thioester bond (Spy0125-CTR^Cys426Ala^) or one or both of the intramolecular isopeptide bonds (Spy0125-CTR^Asp595Ala^, Spy0125-CTR^Asn715Ala^, and Spy0125-CTR^Asp595Ala/Asn715Ala^). We show that the overall secondary structure of the mutant proteins, as observed by CD spectroscopy, is unchanged in all variants when compared to the wild type. We present the results of proteolytic and thermal stability assays showing that the intramolecular isopeptide bonds are the most important for the overall stability of the Spy0125-CTR protein. Furthermore, we have determined the crystal structure of a Spy0125-CTR variant in which the internal thioester is removed. This structure is essentially identical to wild type. Together, these results show that the internal thioester's primary role is unlikely to be to increase protein stability, and it presumably has another role, most likely in mediating pilus adhesion to as-yet unidentified targets on host cells.

## MATERIALS AND METHODS

### Protein cloning, expression, and purification

The initial cloning of Spy0125 residues Asn286-Thr723 (Spy0125-CTR) has been described elsewhere.^20^ For this study, we sub-cloned the same coding region into pOPIN-F.^24^ Spy0125-CTR in pOPIN-F was used as a template for construction of mutants Spy0125-CTR^Cys426Ser^, Spy0125-CTR^Cys426Ala^, Spy0125-CTR^Asn715Ala^, Spy0125-CTR^Asp595Ala^, and Spy0125-CTR^Asp595Ala/Asn715Ala^. These mutations were generated by outsourcing to Genscript and were verified by sequencing. All Spy0125-CTR proteins were overexpressed in *Escherichia coli* BL21 (DE3) grown in Luria Broth at 37°C and induced at *A*_600_ ∼0.4–0.6 with 1 m*M* isopropyl 1-thio-β-d-galactopyranoside. Cells were grown for a further 3 h at 37°C before harvesting by centrifugation. Cell pellets were resuspended in 50 m*M* Tris-HCl, 500 m*M* NaCl, 50 m*M* glycine, 5% (v/v) glycerol, 20 m*M* imidazole, pH 8.0, supplemented with one EDTA-free protease inhibitor cocktail tablet (Roche) per 40 mL of buffer. Cells were lysed by sonication and the lysate clarified by centrifugation. The supernatant was applied to a Ni^2+^-immobilized metal ion affinity chromatography column (GE Healthcare) and proteins were eluted with an imidazole gradient. Fractions containing Spy0125-CTR proteins were pooled and concentrated and then injected onto a Hi-Load 26/60 Superdex 75 gel filtration column (GE Healthcare) pre-equilibrated with 20 m*M* HEPES, 150 m*M* NaCl, pH 7.5. Fractions containing purified Spy0125-CTR proteins were pooled and concentrated to ∼10 mg/mL, as determined by *A*_280_.

### Proteolysis and mass spectrometry analyses

25 μL of 10 mg/mL Spy0125-CTR proteins were added to 50 μL of a solution containing 7.25 μg/mL trypsin in 50 m*M* Tris-HCl, 50 m*M* KCl, 5 m*M* DTT, 5 m*M* MgCl_2_, 10% glycerol. The solutions were incubated for 6 h at room temperature. After incubation, 10 μL of the digest was mixed with 10 μL SDS-PAGE loading dye and heated for 10 min. The samples were then separated on 15% SDS-PAGE gels.

For identification of fragments produced by limited proteolysis, gel bands were excised and further digested with trypsin. The resulting peptides were analyzed by MALDI mass spectrometry and database searches with Mascot defined the regions of the full-length proteins contained within the gel bands.

### Differential scanning fluorimetry

For analysis of thermal denaturation by differential scanning fluorimetry (DSF), Spy0125-CTR proteins were diluted to 1 mg/mL in 50 m*M* di-potassium phosphate, 25 m*M* NaCl, pH 7.2. A total reaction volume of 50 μL comprised 5 μL protein, 5 μL 25× Sypro Orange (Invitrogen), and 40 μL reaction buffer (50 m*M* di-potassium phosphate, 25 m*M* NaCl, pH 7.2). Each experiment was repeated six times in a 96-well plate using a Bio-Rad CFX96 thermal cycler, with a temperature gradient of 20–95°C at steps of 1°C/min. Total fluorescence was detected at every 0.2°C interval. The raw fluorescence data were converted to the differential of the fluorescence divided by the differential of the temperature, and plotted against temperature. The minimum of each curve (inflection point of the fluorescence curve) indicates the melting point (*T*_m_) of each protein.

### CD spectroscopy

CD spectroscopy experiments were performed using a Chirascan-Plus CD spectrophotometer (Applied Photophysics). Purified proteins were buffer exchanged into 50 m*M* di-potassium phosphate, 25 m*M* NaCl, pH 7.2, and concentrated to ∼10 mg/mL by ultrafiltration and then diluted to a concentration of 0.18 mg/mL in 20 m*M* di-potassium phosphate, pH 7.2. CD measurements were carried out in a quartz glass cell with a 0.5-mm path length. To obtain overall CD spectra, wavelength scans between 190 and 260 nm were collected at 20°C using a 2.0-nm bandwith, 0.5-nm step size, and time per point of 1 s. The data were collected over four accumulations and averaged. The raw data in millidegree units were corrected for background and converted to mean residue molar ellipticity. Secondary structure assignments were made using the DichroWeb server, employing the Cdsstr method with reference set 7.^25^–^27^

To obtain thermal melt curves, wavelength scans between 195 and 260 nm were collected using a 2.0-nm bandwidth, 1-nm step size, and time per point of 0.7 s. The temperature was increased from 20 to 90°C at a rate of 1°C/min and a complete scan was collected at 1°C intervals. The raw data, in millidegree units, from 201 to 260 nm, at each temperature measured, were analyzed using the Global 3 software package (Applied Photophysics). The Global 3 package fits the full-spectrum data using the nonlinear regression method of Marquardt and Levenberg^28^ to generate a global analysis of unfolding. This procedure determines fitted and optimized temperatures of transition (melting points: *T*_m_) and their associated Van't Hoff enthalpies (Δ*H*).

### Crystallization, data collection, structure determination, and refinement

Crystals for the Spy0125-CTR^Cys426Ala^ variant were obtained from the same crystallization conditions as the wild type protein, in the *P*2_1_2_1_2_1_ form.^10^ The mother liquor served as a cryo-protectant solution. Diffraction data were collected at beamline I03 at the Diamond Light Source, Oxford, UK. The diffraction data were processed using iMOSFLM^29^ and scaled and merged with SCALA,^30^ as implemented in the CCP4 suite.^31^

The structure was determined by molecular replacement using PHASER^32^ with search models derived from native Spy0125-CTR (PDB: 2XIC). For structure solution the native Spy0125-CTR was divided into two models, one comprising residues Thr290–Val559 and the second residues Ile600–Pro719. The solutions were then combined and refined against the data using REFMAC5.^33^ The final model was produced through iterative rounds of refinement using REFMAC5 and manual rebuilding with COOT.^34^ The final structure comprises residues Thr290–Pro719 in chain A (with the exception of Val319–Ser321 and Ile373–Lys376) and Thr291–Pro719 in chain B (with the exception of Val319–Ser321 and Ile372–Lys376). Translation-Liberation-Screw (TLS) and Noncrystallographic symmetry (NCS) restraints were used in refinement with the top, middle, and bottom domains of the protein forming separate groups. Final structure validation was carried out using MOLPROBITY^35^ and COOT. All data collection and refinement statistics are given in Table1. The atomic coordinate and structure factor files have been deposited in the Protein Data Bank with accession number 4BUG.

**Table 1:** X-Ray Data Collection and Refinement Statistics

Data collection	
Beamline	DLS-I03
Wavelength (Å)	0.976
Space group	P2_1_2_1_2_1_
Resolution range (Å)^a^	70.51–2.80 (2.95–2.80)
Unit cell parameters (Å, ^o^)	*a* = 45.96, *b* = 116.86, *c* = 176.85
	*α* = *β* = *γ* = 90
No. of observations^b^	189,712 (27,915)
Unique reflections^b^	24,374 (3486)
Multiplicity^b^	7.8 (8.0)
<*I*/*σ*(*I*)>^b^	13.0 (4.3)
Completeness (%)^b^	99.9 (100)
*R*_m*erge*_(%)^b^	10.7 (50.3)
Refinement	
Resolution range (Å)^a^	70.51–2.80 (2.95–2.80)
R_*cryst*_ (%)^a^	21.1 (30.8)
R_*free*_ (%)^a^	26.8 (32.9)
Rmsd bond lengths (Å)	0.010
Rmsd bond angles (^o^)	1.33
Average B factor (Å^2^)	
Protein chain A	55.7
Protein chain B	78.5
Water	39.6
Overall MolProbity score^c^	1.88
MolProbity percentile^c^	99th
Ramachandran outliers (%)^c^	0

a Values in parentheses are those for the highest resolution shell.

b Values as reported by SCALA (CCP4).^30^,^31^

c As reported by MolProbity.^35^

## RESULTS

### Variant generation and CD spectra of Spy0125-CTR proteins

To study the role of the intramolecular isopeptide and internal thioester bonds in the stability of Spy0125-CTR, we generated a series of variants that removed the capacity for bond formation. These included mutations to residues Asp595 and Asn715 (both to Ala) that remove single intramolecular isopeptides, and also the double mutant (Asp595Ala/Asn715Ala). To remove the internal thioester, we generated both Cys426Ala and Cys426Ser mutations. All proteins behaved similarly during expression and purification.

**Figure 2:**
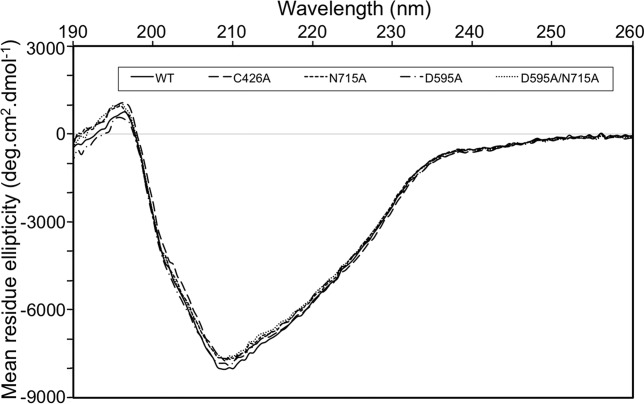
Far-UV CD spectrum of wild type and variants of Spy0125-CTR. Each of the Spy0125-CTR variants adopts an essentially identical structure to the wild type protein, as assayed by Far-UV CD. Amino acids are referred to by their single letter codes.

First, we analyzed the overall secondary structure properties of Spy0125-CTR in solution by CD spectroscopy. The CD spectrum for the native protein displays a negative peak at 209 nm and a positive peak at 195 nm, indicative of a nonclassical β_II-type_ protein (Fig.2). Analysis of the spectrum with Cdsstr is consistent with the crystal structure, giving an α-helical content of 9% and β-sheet content of 32% (Table2, 11 and 36%, respectively, in the crystal structure^10^).

**Table 2:** Secondary Structure Assignment From CD

	2XIC/2XID	WT	Cys426Ala	Asn715Ala	Asp595Ala	Asp595Ala/Asn715Ala
Helix 1		0.03	0.03	0.03	0.03	0.03
Helix 2		0.06	0.05	0.05	0.05	0.05
Total helix	0.11/0.12	0.09	0.08	0.08	0.08	0.08
Strand 1		0.21	0.20	0.19	0.19	0.19
Strand 2		0.11	0.10	0.10	0.10	0.10
Total strand	0.36/0.33	0.32	0.30	0.29	0.29	0.29
Turns		0.22	0.21	0.21	0.21	0.21
Unordered		0.37	0.41	0.42	0.42	0.42
Total other		0.59	0.62	0.63	0.63	0.63
Nrmsd		0.034	0.034	0.036	0.031	0.031

Figures represent fractions of the proteins that comprise each secondary structure element.

Helix 1 and Strand 1 refer to regular α-helices and β-strands, respectively.

Helix 2 and Strand 2 refer to distorted α-helices and β-strands, respectively.^27^ Nrmsd is the normalized root mean squared deviation and represents the goodness of fit of the result to the raw data.

We then analyzed the overall secondary structure properties of Spy0125-CTR variants using the same technique. Each of the variants displayed essentially identical CD spectra to that of the wild type protein (Fig.2). Secondary structure analysis revealed an α-helical content of 8% and a β-sheet content of 29/30% for all variants. This indicates that formation of the intramolecular isopeptide or internal thioester bonds are not critical for maintaining the overall fold of the protein.

### Proteolytic stability of Spy0125-CTR proteins

Next, we investigated whether the intramolecular isopeptide or internal thioester bonds contributed to proteolytic stability of Spy0125-CTR by incubating both the wild-type protein and all variants with trypsin.

**Figure 3:**
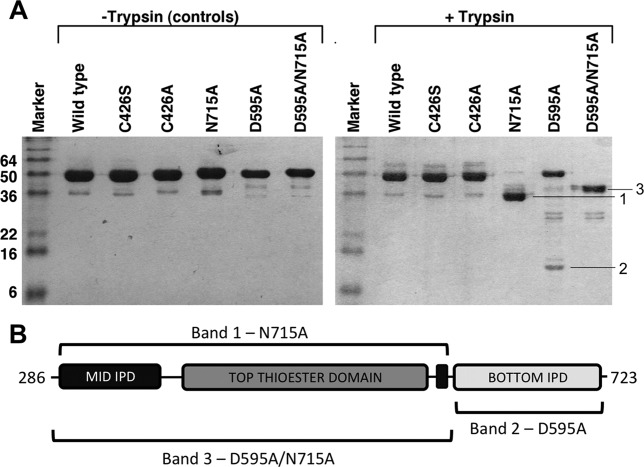
Proteolytic stability of wild type and variants of Spy0125-CTR determined by SDS-PAGE and LC-MS analysis. (**A**) Samples of wild type and variant Spy0125-CTR proteins were incubated with trypsin prior to separation of digestion products by SDS-PAGE. (**B**) Bands 1 (from N715A lane), 2 (from D595A lane) and 3 (from D595A/N715A lane) were excised and analyzed by tryptic digest LC-MS. The regions of the protein covered by peptides in the mass spectrum are indicated on the schematic figure of Spy0125-CTR. IPD, isopeptide domain. Amino acids are referred to by their single letter codes.

Trypsin digests were performed at 37°C for 6 h, and analyzed by SDS-PAGE (Fig.3). The wild type protein remained completely intact after 6 h incubation, indicating a high level of resistance to trypsin cleavage. This high level of resistance to trypsin was also observed for Spy0128.^16^ Interestingly, tryptic digests of Spy0125-CTR variants where the internal thioester has been removed (Spy0125-CTR^Cys426Ala^ and Spy0125-CTR^Cys426Ser^) also revealed intact protein, comparable to wild type. Therefore, the lack of the internal thioester bond in Spy0125-CTR does not confer sensitivity to digestion with trypsin. In stark contrast, the variants where either one or both intramolecular isopeptide bonds were removed (Spy0125-CTR^Asp595Ala^, Spy0125-CTR^Asn715Ala^, and Spy0125-CTR^Asp595Ala/Asn715Ala^) all displayed increased susceptibility to the protease, as visualized by the presence of new bands of lower molecular mass on SDS-PAGE gels (Fig.3).

To identify the regions of the proteins present in the new bands, they were excised from the gels, fully digested with trypsin and subjected to MALDI mass spectrometry. Mascot was then used to map the peptides onto the full-length sequence. For the Spy0125-CTR^Asn715Ala^ variant, which removes the intramolecular isopeptide bond from the Spy0125-CTR bottom domain (Fig.3), the MALDI analysis revealed the remaining band (band 1) comprises the Spy0125-CTR top and middle domains only, with peptides for the bottom domain essentially absent. Therefore, removal of the intramolecular isopeptide bond from the bottom domain renders it susceptible to complete proteolytic degradation with the middle/top domains remaining resistant. For the Spy0125-CTR^Asp595Ala^ variant, which removes the intramolecular isopeptide bond from the Spy0125-CTR middle domain, SDS-PAGE shows that a significant proportion of the protein remains full-length, while there is also some evidence for an intact and stable middle/top domain fragment (faint gel bands). MALDI analysis on the lowest molecular weight band (band 2) reveals that this comprises the Spy0125-CTR bottom domain only. Remarkably, the bottom domain, although cleaved from the full-length protein, remains in itself highly resistant to trypsin, presumably due to the presence of its intact intramolecular isopeptide bond. This shows that removal of the intramolecular isopeptide bond from the middle domain renders the protein prone to partial proteolysis. This allows limited cleavage of the bottom domain from the full-length protein, but leaves the bottom domain and top/middle domain fragments in themselves resistant. For the double variant (Spy0125-CTR^Asp595Ala/Asn715Ala^), which removes both the intramolecular isopeptide bonds, the results from the single mutants are essentially compounded, the bottom domain is fully cleaved and completely digested but the top/middle domains remain largely intact (band 3, Fig.3). There is a small difference in apparent molecular mass of bands 1 and 3 (Fig.3) on the gel although MALDI analysis of both of these bands reveals they cover the top and middle domains. To date, we have not been able to determine the subtle differences between the fragments in these bands.

These results show that susceptibility of Spy0125-CTR to limited proteolysis is conferred by the mutation of the intramolecular isopeptide bonds in the protein but not mutation of the internal thioester bond.

### Thermal stability of Spy0125-CTR and its variants assayed by differential scanning fluorimetry

**Figure 4:**
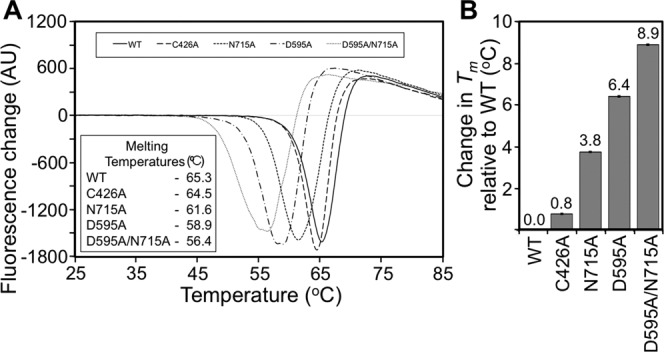
Thermal stability of Spy0125-CTR and its variants assayed by differential scanning fluorimetry. (**A**) First derivative curves of thermal denaturation data for wild type and Spy0125-CTR variants reveal the melting temperatures (inset table) for each protein. (**B**) A bar chart representation of the changes in melting temperature observed for each Spy0125-CTR variant compared to wild type. Amino acids are referred to by their single letter codes.

To assess the thermal stability of the Spy0125-CTR proteins, denaturation assays were performed using DSF (Fig.4). DSF measures global unfolding of a protein through the binding of a fluorescent dye to hydrophobic surfaces. Using this technique, the wild type protein gave a single unfolding transition with a melting temperature (*T*_m_) of 65.3°C, indicative of a well-folded, stable protein [Fig.4(A)]. The internal thioester variant (Spy0125-CTR^Cys426Ala^) also gave a single unfolding transition with a *T*_m_ of 64.5°C, a small decrease of 0.8°C compared to the wild type (Fig.4). To study the internal thioester, we only used the Spy0125-CTR^Cys426Ala^, and not the Spy0125-CTR^Cys426Ser^, in all thermal denaturation assays, as this is the mutant that has been characterized in the host bacteria and is therefore of most relevance. In contrast to Spy0125-CTR^Cys426Ala^, all three intramolecular isopeptide bond variants caused a significant decrease in *T*_m_ (all single transitions) with respect to the wild type protein (Fig.4). Removal of the bottom domain intramolecular isopeptide (Spy0125-CTR^Asn715Ala^) caused a decrease of 3.7°C, (*T*_m_ = 61.6°C), while removal of the middle domain intramolecular isopeptide (Spy0125-CTR^Asp595Ala^) caused a larger decrease of 6.4°C (*T*_m_ = 58.9°C). This negative shift in *T*_m_ is compounded in the double intramolecular isopeptide variant (Spy0125-CTR^Asp595Ala/Asn715Ala^), resulting in a decrease of 8.9°C (*T*_m_ = 56.4°C).

### Thermal stability of Spy0125-CTR and its variants assayed by circular dichroism

In addition to measuring protein unfolding by DSF, we monitored the thermal denaturation of Spy0125-CTR proteins using CD spectroscopy. We reasoned that this technique might detect more subtle structural changes, possibly missed by DSF, which could be correlated with the mutations introduced to the variant proteins.

At each 1°C interval during the temperature ramp (between 20 and 90°C), a CD spectrum was collected between 195 and 260 nm (Supporting Information Fig. S1). All data for each wavelength at each temperature were converted to the differential of the millidegree units divided by the differential of the temperature, and plotted against temperature (first derivative curve), using the Global3 analysis software (Applied Photophysics) to estimate initial *T*_m_ and Van't Hoff Δ*H* values for the nonlinear regression fitting procedure. The Global3 software was then used to calculate a fitted *T*_m_ and associated Van't Hoff Δ*H* for each unfolding transition (Table3). We also tested the reversibility of unfolding by cooling samples back to 20°C after heating (Supporting Information Fig. S2). Each of Spy0125, Spy0125-CTR^Cys426Ala^, and Spy0125-CTR^Asp595Ala^ return to very similar CD traces at 20°C following heating; Spy0125-CTR^Asn715Ala^ and Spy0125-CTR^Asp595Ala/Asn715Ala^ do not appear to fully return to their native conformations.

**Table 3:** *T*_m_s and Van't Hoff Parameters From Fitting Thermal Denaturation CD Spectra Using the Global 3 Software

	Lower temperature transition		Higher temperature transition	
	*T*_m_ (°C)	Van't Hoff ΔH (kJ/mol)	*T*_m_ (°C)	Van't Hoff ΔH (kJ/mol)
WT	66.9 ± 0.1	723.7 ± 37.0	78.2 ± 0.1	473.5 ± 7.6
C426A	65.2 ± 0.1	473.5 ± 10.9	78.4 ± 0.1	516.4 ± 6.1
N715A	64.3 ± 0.1	614.5 ± 19.2	50.3 ± 0.1	359.5 ± 5.4
D595A	61.0 ± 0.1	451.1 ± 13.1	78.4 ± 0.1	480.7 ± 5.9
D595A/N715A	58.0 ± 0.2	259.9 ± 9.6	49.9 ± 0.1	298.7 ± 5.6

**Figure 5:**
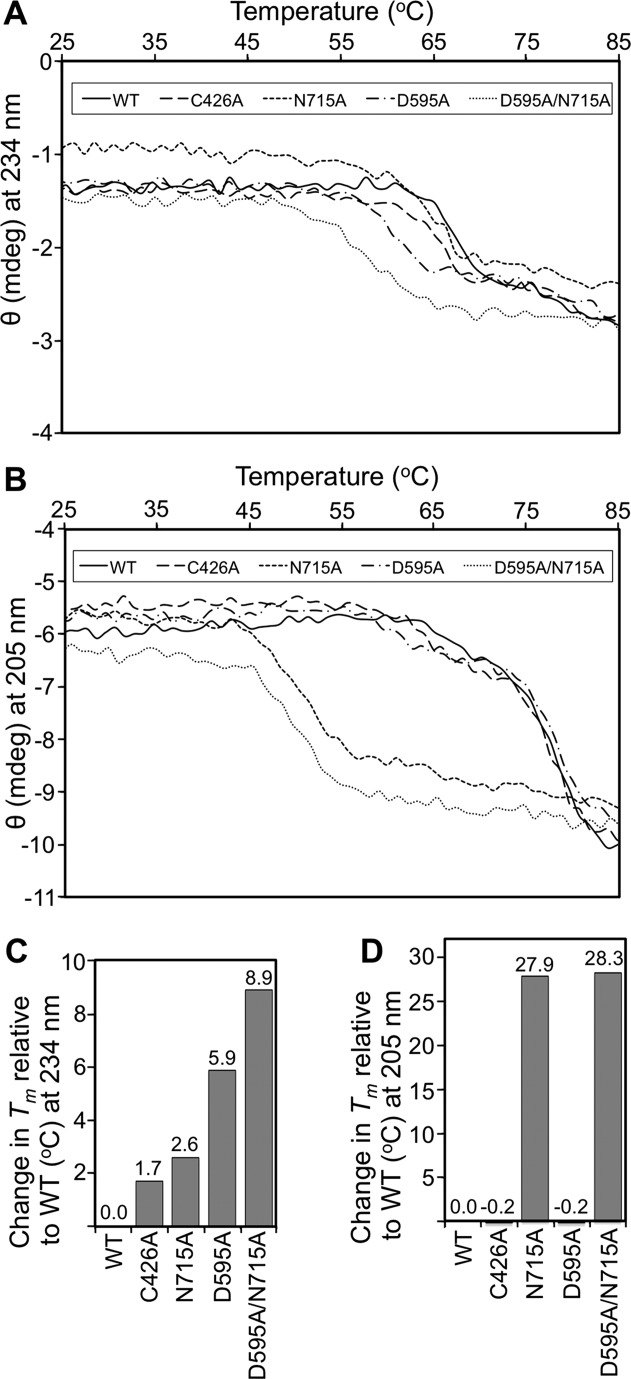
Thermal stability of Spy0125-CTR and its variants assayed by circular dichroism. Thermal denaturation data for wild type and Spy0125-CTR variants visualized by CD at 234 nm (lower temperature transition, **A**) and 205 nm (higher temperature transition, **B**). (**C**) and (**D**) Bar chart representations of the changes in melting temperature observed for each Spy0125-CTR variant compared to wild type at the given wavelength and transition monitored. Amino acids are referred to by their single letter codes.

For each of the five proteins, we observed two distinct transitions. When the raw spectral data (Supporting Information Fig. S1) are plotted as a function of temperature at each wavelength, the individual unfolding transitions (calculated from the global data) were most readily identifiable at wavelengths of 234 nm (lower temperature transition) and 205 nm (higher temperature transition). Therefore, spectra at these specific wavelengths were separately plotted, for ease of visualizing the transitions, but they are representative of the overall global unfolding analysis (Fig.5 and Supporting Information Fig. S3). While signatures of both transitions can be seen at both 205 and 234 nm, one of the transitions is most distinct at each of these wavelengths (Fig.5 and Supporting Information Fig. S3). For the wild-type protein, the melting temperatures for the two transitions are 66.9°C, predominantly observed at 234 nm, and 78.2°C, seen at 205 nm (Fig.5). The *T*_m_ at 66.9°C correlates well with the *T*_m_ measured by DSF (65.3°C, Fig.4).

The internal thioester bond variant (Spy0125-CTR^Cys426Ala^) showed very similar transitions to the native protein with *T*_m_s of 65.2 and 78.4°C, visualized by the spectra at 234 nm and 205 nm respectively (Fig.5). Therefore, the transition at ∼78°C is unaffected by this mutation (Fig.5), whereas the lower temperature transition has decreased very slightly (1.7°C, Fig.5). The transition at the lower temperature is also consistent with the DSF analysis, which showed a very small decrease (0.8°C) in *T*_m_ for this variant (Fig.4). Interestingly, the calculated Van't Hoff Δ*H* for this transition is 473.5 kJ/mol, compared with 723.7 kJ/mol for the wild type.

For the bottom domain intramolecular isopeptide variant (Spy0125-CTR^Asn715Ala^), we observed a 2.6°C decrease in *T*_m_ for the lower temperature transition_,_ visualized at 234 nm (64.3°C), compared to the native protein (Fig.5). This is similar to the 3.7°C shift seen by DSF. Interestingly, the *T*_m_ of the higher temperature transition, best observed at 205 nm, decreased from 78.2 to 50.3°C, a remarkable shift of 27.9°C derived from the introduction of a single, subtle mutation (Fig.5). This change is similar to the result of an equivalent mutation in the Spy0128 protein, where a 34°C shift was obtained.^16^ Given the dramatic decrease in the higher *T*_m_ transition with this mutation, we attribute it to the bottom domain only. It appears that this mutation only subtly affects the other domains, as observed by the 2.6°C *T*_m_ shift for the first transition (64.3°C, Fig.5). We therefore predict that the lower *T*_m_ transition is predominantly reporting on the unfolding of the top/middle domains. A single *T*_m_ for both these domains is not surprising given that the top domain is an insertion into the middle domain and intimate contacts are maintained between these regions.^10^

For the middle domain intramolecular isopeptide variant (Spy0125-CTR^Asp595Ala^), we observed a 5.9°C decrease in the lower *T*_m_ transition (visualized at 234 nm, 61.0°C, Fig.5). Again, this is consistent with the DSF data, which showed a 6.4°C decrease for this variant. In contrast to the Spy0125-CTR^Asn715Ala^ variant, essentially no change was observed for the higher *T*_m_ transition (best observed at 205 nm, 78.4°C, Fig.5). This further supports the hypothesis that the higher *T*_m_ transition is reporting on the unfolding of the bottom domain and the lower *T*_m_ transition reporting on the unfolding of the top/middle domains.

For the double intramolecular isopeptide variant (Spy0125-CTR^Asp595Ala/Asn715Ala^), significant shifts in the transitions visualized at both 234 nm (*T*_m_ of 58°C) and at 205 nm (*T*_m_ of 49.9°C) are observed (Fig.5). The change seen for the higher temperature transition correlates well with the single Spy0125-CTR^Asn715Ala^ (a shift of 28.3°C in the double mutant). For the lower temperature transition, we observe a large 8.9°C decrease in the *T*_m_. Again, mutation of the intramolecular isopeptide in the bottom domain appears to cause an increased destabilization of the top/middle domains.

The thermal unfolding data derived from both DSF and CD show that the intramolecular isopeptide bonds within the middle and bottom domains of Spy0125-CTR confer significant thermal stability to the protein. Overall, the internal thioester does not appear to be as relevant for stability.

### Crystal structure of the Spy0125-CTR^Cys426Ala^ variant lacking the internal thioester bond

Removal of the internal thioester bond from Spy0125 results in an ∼75% decrease in pilus-mediated adhesion to model HaCaT cells, compared to wild type.^10^ The results of the protease and thermal stability assays suggest this cannot be due to misfolding, or instability of the internal thioester variant protein. To establish whether there are any minor conformational differences between the native and internal thioester variant proteins that could cause the reduction in adhesion (rather than just the loss of the internal thioester), we determined the crystal structure of the Spy0125-CTR^Cys426Ala^ variant.

**Figure 6:**
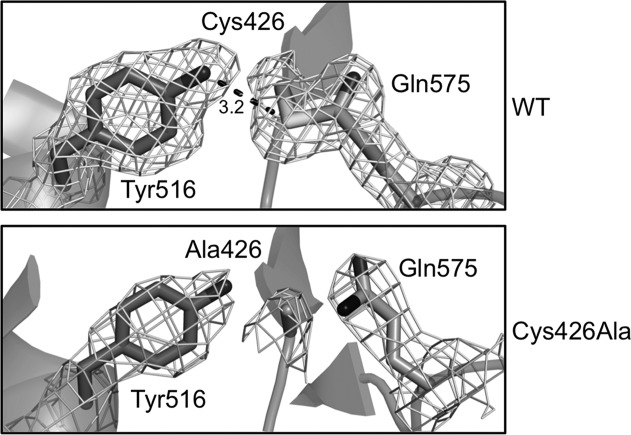
X-ray structure of Spy0125-CTR^Cys426Ala^ confirms removal of the internal thioester does not significantly affect the local protein structure. Electron density in the vicinity of the internal thioester for Spy0125-CTR^10^ and Spy0125-CTR^Cys426Ala^. 2*mF*_obs_-*DF_calc_* electron density maps (calculated by Refmac5) are contoured at 1.0*σ*.

The structure shows that the overall fold of the variant is essentially identical to that of the native protein in the *P*2_1_2_1_2_1_ crystal form.^10^ The root mean square deviation (rmsd) between the Cα atoms of chain A of the Spy0125-CTR^Cys426Ala^ structure and chain B of the native is 0.65 Å. The Spy0125-CTR^Cys426Ala^ structure displays the same overall secondary structural elements, with slight differences only apparent in flexible loop regions. The electron density defining the position of Ala426 and Gln575 shows no link between these side chains, as expected (Fig.6). A small reorientation of the side chain of Gln575 results in a new hydrogen bond between the Gln575^NE2^ atom and the carbonyl O of Asn428. Tyr516, that is in close proximity to the internal thioester in the native structure is located in the same orientation, but no longer forms any hydrogen bonding interaction with these residues.

The structure reveals that the only consequence of the Cys426Ala mutation on the protein is the removal of the internal thioester. There are no significant rearrangements in the overall protein fold that can explain the ∼75% loss of bacterial binding previously observed in the adhesion assay.

## DISCUSSION

The mechanisms of how pili are assembled on the surface of Gram-positive bacteria, and how they mediate interaction with host cells to bring about infection, have been studied intensely since the molecular details of their composition was first defined in 2005.^8^,^36^ In M1 Group A *Streptococcus* strain SF370, the major pilin subunit Spy0128, which makes up the polymeric backbone of the pilus, contains strategically positioned intramolecular isopeptide bonds in each of its two β-sandwich domains.^9^ These bonds have been shown to confer proteolytic, thermal and mechanical stability to the protein.^16^,^17^ Similar experiments with other intramolecular isopeptide bond containing domains have shown similar results.^13^,^15^,^37^ These properties likely allow the proteins to remain stable and endure the stresses associated with the harsh environments at the sites of infection.

In this study, we have shown that the positive contribution of these strategically placed intramolecular isopeptide bonds to protein stability is not limited to the backbone proteins of pili, but can also be important for the adhesins presented at the pilus tip, in this case Spy0125. We also show that intramolecular isopeptide bonds are not required for the overall folding of the Spy0125 protein *in vitro*. To ensure as little disruption to the fold as possible, we made mutations to the Asp (595) and Asn (715) residues that are each one of the amino acids linked in the isopeptide bonds. Kang and Baker showed that mutations to these residues are the least disruptive to the hydrophobic core of the β-sandwich domains in Spy0128 (when compared to mutations in either the Lys (that forms the bond with an Asn) or Glu (that catalyzes bond formation)), and the surrounding structure is virtually undisturbed.^16^ While we have not obtained crystal structures of the Spy0125-CTR^Asp595Ala^, Spy0125-CTR^Asn715Ala^, or Spy0125-CTR^Asp595Ala/Asn715Ala^ variants, we predict that in the absence of isopeptide bond formation, Glu347 and Lys297 and Glu680 and Lys610 interact to form hydrogen bonds in each of the two Spy0125-CTR β-sandwich domains.

To study the contribution of the internal thioester bond to protein stability we made the Cys426Ala mutation as this variant would cause the least disruption to the protein fold, rather than mutating Cys426 to any other residue or mutating Gln575. Compared to the isopeptide bond-based mutations, the internal thioester Spy0125-CTR^Cys426Ala^ mutation does not compromise the proteolytic stability, or significantly affect the thermal stability of the protein as measured by the *T*_m_. However, we note that the Van't Hoff Δ*H* for the lower temperature transition is reduced for this mutant compared to wild type. This 35% reduction shows that the internal thioester does have some measureable contribution to protein stability even if this is not detectable by trypsin proteolysis or obvious from the *T*_m_ monitored during thermal denaturation. As this mutation removes a covalent link between distant parts of the protein it is perhaps not surprising that removing it has some measurable effect, although taken together with our other data this is not as significant as the mutations removing the isopeptide bonds. Showing that the overall CD spectrum, proteolytic/thermal stability, and crystal structure of Spy0125-CTR^Cys426Ala^ are similar to wild-type protein provides additional, albeit indirect, evidence that the internal thioester has another function—most likely in mediating adhesion. Although, how this bond is involved in promoting adhesion to cells, and what the identity of the molecular players are at the host cell interface, awaits further study.

In recent years, DSF has become a popular tool for assessing protein stability. For example, it can be used to define buffer conditions in which a protein is most stable, or can be used to assay ligand binding.^38^,^39^ It is a highly appropriate method to use in simple systems when a large number of conditions, or large number of samples, need to be screened as it is easily adapted to miniaturization and repetition. However, in more complicated systems, significant information may be lost. Interestingly, DSF was used to investigate the stability of the RrgA subunit of the *rlr* pilus in *S. pneumoniae*.^13^ Although the fluorescence was dominated by a single unfolding transition (at ∼51°C), two maxima in the signal were obtained for this four-domain protein. The weak second transition (at ∼64°C) could be associated with presence/absence of intramolecular isopeptide bonds.^13^ Here, we used two independent methods to study the thermal unfolding of Spy0125-CTR and the variants: DSF and circular dichroism. By DSF, we observed changes in an, apparent, single thermal unfolding transition of Spy0125-CTR that positively correlated with the loss of intramolecular isopeptide bonds, but not with the loss of the internal thioester. Interestingly, by monitoring thermal unfolding by CD, across the far-UV spectrum from 195 to 260 nm, we identified two transitions that could be directly associated with mutations in the individual β-sandwich domains of Spy0125-CTR. This observation highlights the importance of using different experimental approaches to monitor protein stability *in vitro*, in order to uncover biological function. This is perhaps especially important in samples where multiple protein domains are present.
