# Supplementary material for: Intramolecular isopeptide but not internal thioester bonds confer proteolytic and significant thermal stability to the S. pyogenes pilus adhesin Spy0125
Source: Proteins. 2013 Oct 17;82(3):517–27. doi: 10.1002/prot.24420 (PMC4282584; doi:10.1002/prot.24420)
Supplement: Supplementary file 1 — Supplementary Information [file prot0082-0517-SD1.docx]

**SUPPLEMENTARY INFORMATION**

**Intramolecular isopeptide but not internal thioester bonds confer proteolytic and significant thermal stability to the *S. pyogenes* pilus adhesin Spy0125**

Walden M, Crow A^1^, Nelson MD, Banfield MJ^*^

Dept. of Biological Chemistry, John Innes Centre, Norwich Research Park, Norwich, NR4 7UH, UK

^1^ Present address: Dept. of Pathology, University of Cambridge, Tennis Court Road, Cambridge, CB2 1QP, UK

^*^ Correspondence to MJB. Email: mark.banfield@jic.ac.uk; Phone: +44 (0)1603 450742; Fax: +44 (0)1603 450018


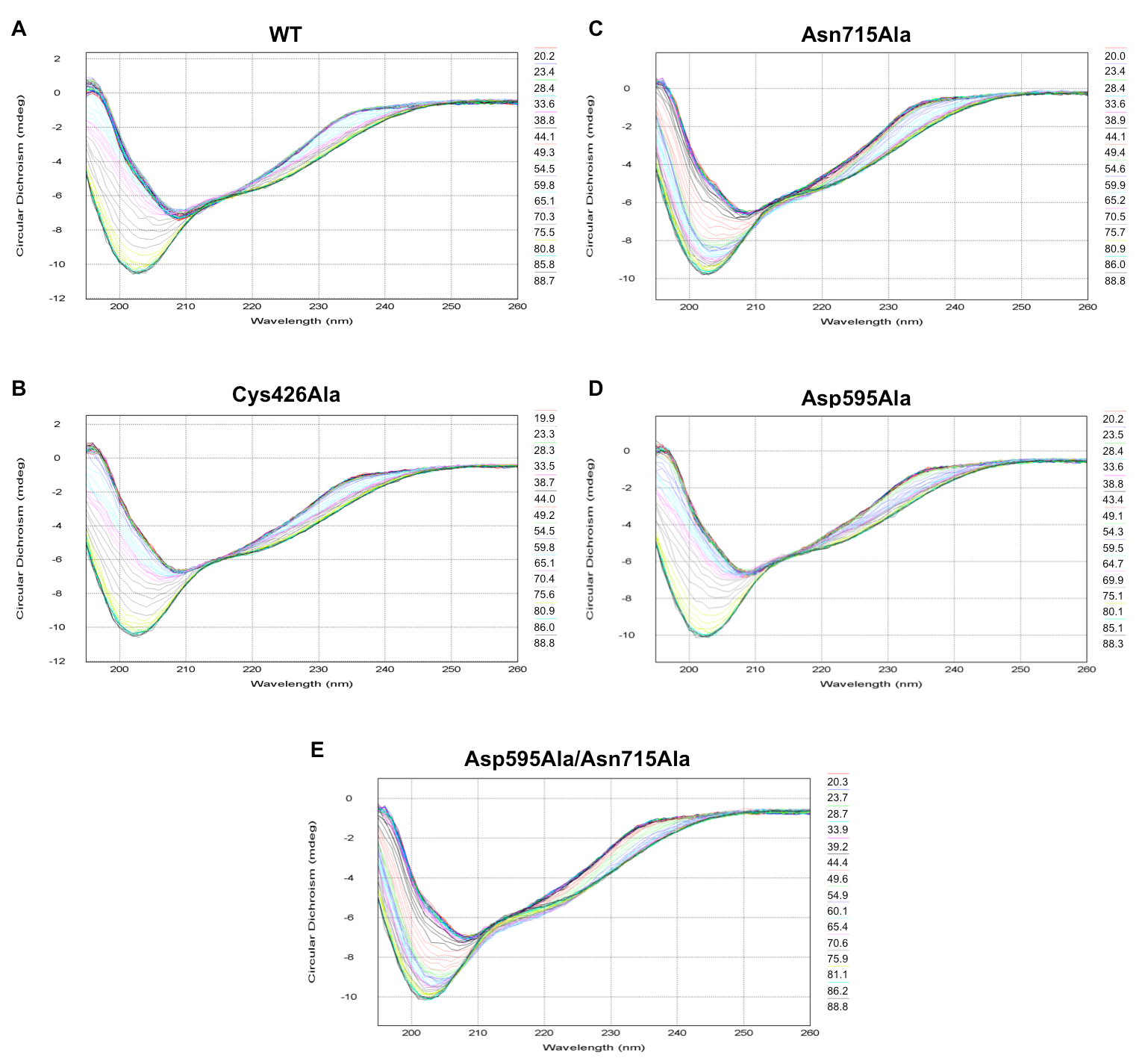


Figure S1: **Thermal unfolding curves of Spy0125-CTR wild type and variant proteins monitored by CD.** CD spectra from 195 – 260 nm were collected as the temperature was ramped from 20 to 90^o^C at a rate of 1^o^C per min. A complete scan was collected at 1^o^C intervals.


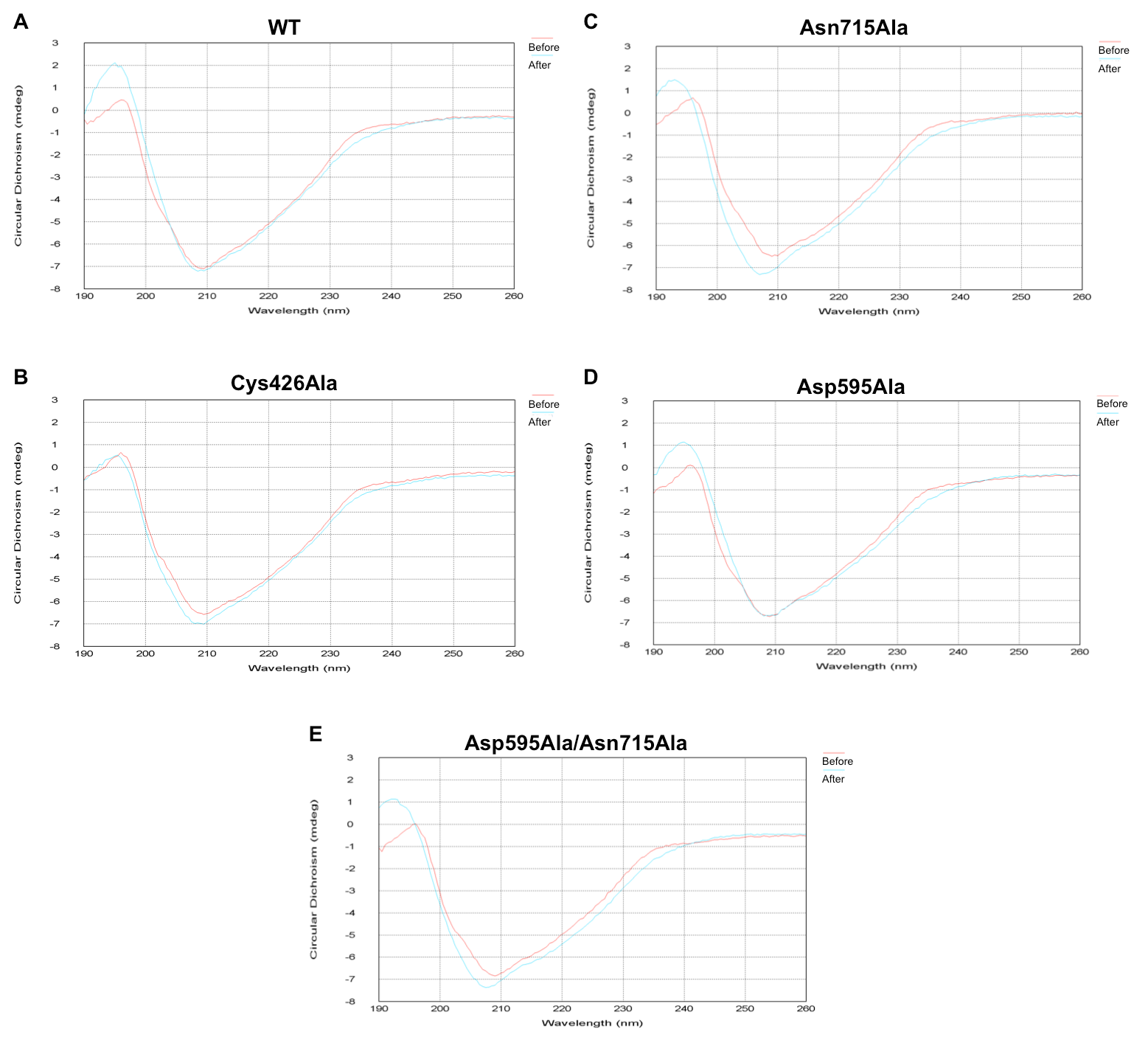


Figure S2: **Reversibility of thermal unfolding monitored by CD.** Red lines represent CD spectra collected for each of the wild type Spy0125-CTR and variants at 20.0^o^C prior to heating. Blue lines are the same spectra collected following heating and returning to 20.0^o^C.


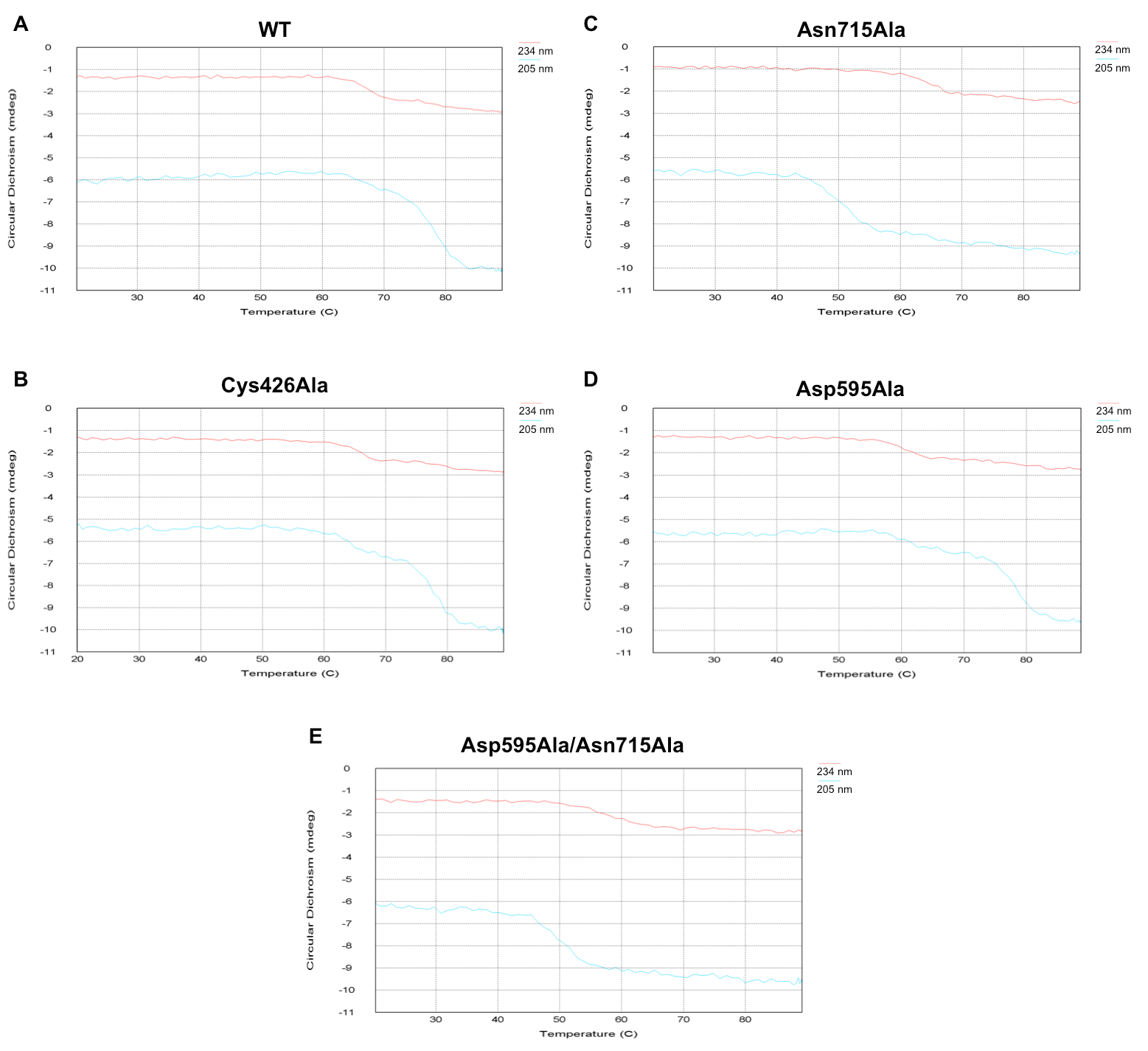


Figure S3: **CD spectra observed at 234 nm and 205 nm during heating.** Individual spectra for each of the wild type Spy0125-CTR and variants at the wavelengths indicated.
